# Vasculitic neuropathy: clinical features, diagnostic approach, and neurological outcomes in a single-center cohort

**DOI:** 10.3389/fneur.2026.1886100

**Published:** 2026-08-18

**Authors:** Sevinch Rakhmonova, Chafic Karam

**Affiliations:** 1 Department of Neurology, Perelman School of Medicine, University of Pennsylvania, Philadelphia, PA, United States; 2 Department of Neurology, Perelman School of Medicine, University of Pennsylvania, Philadelphia, PA, United States

**Keywords:** ANCA-associated vasculitis, cohort study, diabetic lumbosacral radiculoplexus neuropathy, mononeuritis multiplex, neuropathy impairment score, nonsystemic vasculitic neuropathy, vasculitic neuropathy

## Abstract

**Background:**

Vasculitic neuropathy (VN) is a diverse group of immune-mediated peripheral neuropathies that result in systemic or non-systemic vasculitic neuropathy. There are limited studies that characterize the neurological outcome of VN patients. We aimed to describe the clinical features, diagnostic workup, and neurological outcomes of VN patients.

**Methods:**

We retrospectively reviewed adults with VN evaluated at the Hospital of the University of Pennsylvania between October 2020 and December 2025. Diagnostic classification followed the Peripheral Nerve Society 2010 criteria and the 2022 American College of Rheumatology/European Alliance of Associations for Rheumatology classification criteria for systemic vasculitides. Definite vasculitis was defined by biopsy confirmation or fulfillment of formal classification criteria; probable vasculitis was supported by clinical, electrodiagnostic, and serologic features. Neurological impairment was quantified using the Neuropathy Impairment Score (NIS). Improvement was defined *a priori* as a decrease in NIS of at least 2 points (ΔNIS ≤ − 2). Continuous variables were compared using Welch two-sample *t*-tests; categorical variables using Fisher exact tests.

**Results:**

Of 85 patients, 66 met inclusion criteria. Non-systemic disease was analyzed as two distinct entities, the NSVN subtype (*n* = 18, 27.3%) and lumbosacral radiculoplexus neuropathy (LRPN; *n* = 15, 13 diabetic and 2 non-diabetic), alongside systemic vasculitic neuropathy (*n* = 33). The most common subtypes were the NSVN subtype (27.3%), ANCA-associated vasculitis (22.7%), and diabetic LRPN (19.7%). Biopsy was performed in 48 patients and demonstrated vasculitis in 41; the remaining patients met criteria for possible vasculitic neuropathy on clinical, electrodiagnostic, and serologic grounds (59 definite, 7 possible overall). Over a median follow-up of 13.5 months (IQR 6.2–31.2), 39 patients (59.1%) improved by ≥ 2 NIS points, 5 (7.6%) were stable, and 22 (33.3%) worsened. Patients who improved had higher baseline NIS-motor subscores (17.3 ± 14.6 vs. 6.7 ± 7.9, *p* < 0.001) and shorter diagnostic delays (12.3 ± 16.4 vs. 30.3 ± 41.7 months, *p* = 0.042).

**Conclusion:**

Majority of patients experienced neurological improvement on the NIS. Greater baseline motor impairment and shorter diagnostic delay were associated with improvement. These findings characterize the clinical course of VN in a contemporary tertiary-care setting and provide a benchmark for future multicenter studies.

## Introduction

Vasculitic neuropathy (VN) results from inflammation of the vasa nervorum with consequent ischemic injury to peripheral nerves. According to the 2012 revised International Chapel Hill Consensus Conference, vasculitides are classified as primary systemic, secondary systemic, or non-systemic forms confined to a single organ (1). Peripheral nerves are commonly involved across these categories, and VN may be the presenting manifestation of an underlying systemic process or may exist in isolation as non-systemic vasculitic neuropathy (NSVN) (2, 3). In the Peripheral Nerve Society 2010 guideline, the diagnosis of NSVN was operationalized as a clinical, electrodiagnostic, and pathologic syndrome supported by exclusion of systemic disease (4). More recently, the 2022 American College of Rheumatology/European Alliance of Associations for Rheumatology criteria have refined the classification of ANCA-associated vasculitides (5–7).

Clinically, VN is heterogeneous. The classic phenotype is an acute or subacute multifocal painful neuropathy known as mononeuritis multiplex (2), but length-dependent, asymmetric, and radiculoplexus presentations are also recognized. Diabetic and non-diabetic lumbosacral radiculoplexus neuropathies (LRPN), characterized by microvasculitic injury at the level of the nerve roots and plexus, are considered within the non-systemic spectrum (8–10). The recently described entity of vasculitic myopathy further broadens the clinical spectrum of vasculitic involvement of the neuromuscular system (11).

Despite advances in classification and immunomodulatory therapy, contemporary outcome data for VN remain limited. Published cohorts are often small, focused on specific subtypes [e.g., NSVN (3, 12), or LRPN (9, 10)], or report outcomes using non-quantitative scales. The Neuropathy Impairment Score (NIS) is a validated examination-based composite measure of cranial-nerve, motor, sensory, and reflex impairment originally developed and validated in the Rochester Diabetic Neuropathy Study (13), and has since been used as a standard outcome measure in clinical trials and observational studies of peripheral neuropathy. Few series have applied the NIS systematically across the heterogeneous spectrum of VN, and there is no recent United States tertiary-center description of VN outcomes for comparison with European and other international cohorts (12, 14).

The objective of this study was to describe the clinical features, diagnostic workup, treatment patterns, and neurological outcomes of a contemporary cohort of patients with VN evaluated at a single tertiary US neuromuscular referral center. We additionally undertook exploratory analyses to identify clinical factors associated with neurological improvement.

## Methods

### Study design and setting

We performed a retrospective cohort study of adults evaluated for VN at the Hospital of the University of Pennsylvania (HUP) between October 2020 and December 2025. The study was approved by the University of Pennsylvania Institutional Review Board with a waiver of informed consent given the retrospective design.

### Patient selection and diagnostic classification

Eligible patients were adults (≥ 18 years) with a clinical diagnosis of vasculitic neuropathy who had documented baseline and follow-up neurological assessments. Patients were classified as having definite vasculitis if (a) a tissue biopsy demonstrated vessel wall inflammation with vascular damage, or (b) they met the 2022 American College of Rheumatology/European Alliance of Associations for Rheumatology classification criteria for granulomatosis with polyangiitis, microscopic polyangiitis, or eosinophilic granulomatosis with polyangiitis (5–7), or fulfilled established criteria for cryoglobulinemic vasculitis or polyarteritis nodosa (1). The remaining patients were classified as having probable vasculitic neuropathy per the Peripheral Nerve Society 2010 criteria (4), with the diagnosis supported by characteristic clinical features, electrodiagnostic confirmation of an axonal asymmetric or multifocal neuropathy, and serologic findings consistent with vasculitis after exclusion of alternative causes. Patients were further categorized as systemic or non-systemic according to the involvement of organs or tissues beyond the peripheral nervous system. Non-systemic disease comprised NSVN and diabetic lumbosacral radiculoplexus neuropathy, the latter included *a priori* given its established microvasculitic pathophysiology (8, 10). Consistent with the 2010 Peripheral Nerve Society guideline, definite vasculitic neuropathy required biopsy confirmation of vasculitis, whereas patients with characteristic clinical, electrodiagnostic, and serologic features but a non-diagnostic or unperformed biopsy were classified as possible vasculitic neuropathy; both groups were included, as nerve biopsy is unrevealing in approximately half of vasculitic neuropathy cases (19). Because the term “nonsystemic vasculitic neuropathy” is used in the literature both for all non-systemic disease and for a specific phenotypic subtype, we separated lumbosacral radiculoplexus neuropathy (LRPN) from the NSVN subtype throughout. LRPN was analyzed as a distinct non-systemic entity (13 diabetic and 2 non-diabetic patients), reflecting its microvasculitic pathophysiology and its classification within vasculitic neuropathy.

Patients were excluded if (i) they had not been evaluated by Neurology, (ii) documentation was insufficient to score a baseline NIS, (iii) no follow-up assessment was available to score a final NIS, or (iv) an alternative diagnosis was established on chart review.

### Clinical and laboratory data

Demographic, clinical, laboratory, and treatment data were extracted from the electronic medical record using a standardized data abstraction form. Recorded variables included age, sex, vasculitis subtype, neuropathy pattern, baseline and final NIS subscores (motor, sensory, reflex), date of symptom onset, date of initiation of immunomodulatory therapy, and follow-up duration. Diagnostic delay was defined as the time interval (in months) from documented symptom onset to initiation of immunomodulatory therapy. Serologic and laboratory findings (ANCA with myeloperoxidase and proteinase 3 sub-specification, cryoglobulins, complement C4, rheumatoid factor, immunoglobulins M and G, erythrocyte sedimentation rate, C-reactive protein, glycated hemoglobin, and cerebrospinal fluid protein) were recorded when available; results are reported as the number of patients with documented abnormal values divided by the number with non-missing data for that variable.

### Electrodiagnostic studies

All electrodiagnostic studies were performed at the Hospital of the University of Pennsylvania electrodiagnostic laboratory, which is accredited with exemplary status by the American Association of Neuromuscular and Electrodiagnostic Medicine (AANEM) (15). Standardized motor and sensory nerve conduction studies were performed bilaterally, typically including median, ulnar, radial, peroneal, tibial, and sural nerves, with additional nerves sampled based on the clinical pattern (e.g., lateral femoral cutaneous, superficial peroneal, or musculocutaneous nerves for suspected mononeuritis multiplex). Concentric needle electromyography was performed in clinically affected and contralateral asymptomatic limbs to characterize the distribution of motor unit involvement. Neuropathy patterns were classified by the attending neuromuscular neurologist as length-dependent polyneuropathy, asymmetric polyneuropathy, mononeuritis multiplex/multifocal neuropathy, and radiculoplexus neuropathy.

### Diagnostic workflow

At our institution, patients were evaluated using a structured diagnostic workflow. Evaluation began with a clinical history suggestive of vasculitic neuropathy, defined as acute or subacute onset, a multifocal or rapidly progressive course, or a polyneuropathy accompanied by systemic features (skin changes, weight loss, or respiratory, renal, or gastrointestinal symptoms). Patients then underwent a neurological examination and electrodiagnostic studies to confirm features supportive of vasculitic neuropathy. Serologic testing for systemic vasculitis followed, including ANCA, rheumatoid factor, cryoglobulins, and hepatitis serologies, together with tests for extraneural organ involvement (renal function, urinalysis, and chest imaging). When indicated, an affected organ was identified for biopsy, including nerve, particularly in suspected NSVN. A biopsy demonstrating vasculitis confirmed the diagnosis and prompted treatment; when biopsy was non-diagnostic, an additional biopsy was considered, or treatment was initiated when clinical suspicion was high.

### Outcome measures

The primary outcome was the change in Neuropathy Impairment Score (NIS) from baseline to last follow-up. The NIS is a composite examination-based measure of cranial nerve, muscle strength, sensory, and reflex impairment, with higher scores indicating greater impairment (13). Patients were classified as Improved if the change in NIS was ≤ − 2 points, and as Not Improved otherwise. Specifically, a change of exactly −2 was classified as Improved, a change of exactly +2 was classified as Worsened (a subgroup of Not Improved), and a change between −1 and +1 inclusive was classified as Stable (also included within Not Improved for the primary analysis). This dichotomization was pre-specified to reflect a clinically meaningful between-visit change in the NIS.

### Statistical analysis

Continuous variables were summarized as mean ± standard deviation (SD); categorical variables as *n* (%). Between-group comparisons used Welch two-sample *t*-tests for continuous variables and Fisher exact tests for categorical variables (chi-square was used for categorical variables with more than two levels when expected cell counts allowed). Within-patient change in NIS from baseline to follow-up was assessed using the paired *t*-test. Parametric testing was selected throughout, as the Central Limit Theorem supports approximate normality of the sample means at *n* = 66 despite the right-skewed distribution of NIS scores.

Exploratory multivariable logistic regression was performed to identify factors associated with neurological improvement. Candidate predictors were pre-specified on the basis of (i) a-priori clinical and biological rationale (neuropathy pattern category, baseline motor impairment severity, age), (ii) reviewer-recommended classifiers (systemic vs. non-systemic vasculitis, per the Peripheral Nerve Society 2010 criteria), and (iii) variables associated with the outcome at univariate *p* < 0.10 (diagnostic delay). Stepwise selection was not used. Multicollinearity was assessed using variance inflation factors (all < 2.5). Model calibration was evaluated with the Hosmer–Lemeshow goodness-of-fit test, and discrimination with the area under the receiver operating characteristic curve. With 27 events in the smaller outcome group and 5 candidate predictors, the model achieved approximately 5.4 events per variable; multivariable estimates are therefore presented as descriptive associations rather than for clinical prediction (16). No adjustment was made for multiple comparisons given the descriptive purpose of the analysis. *p* values < 0.05 were considered statistically significant. Analyses were performed in R version 4.5.1 (R Foundation for Statistical Computing, Vienna, Austria).

### Missing data and data availability

Data were extracted from a single retrospective spreadsheet. For variables with incomplete documentation in the chart (e.g., serologic and laboratory results, which were not uniformly ordered across patients), results are reported as *n*/*N* (%) where N is the number of patients with non-missing data for that variable. No imputation was performed.

## Results

### Cohort and demographic features

**Figure 1:**
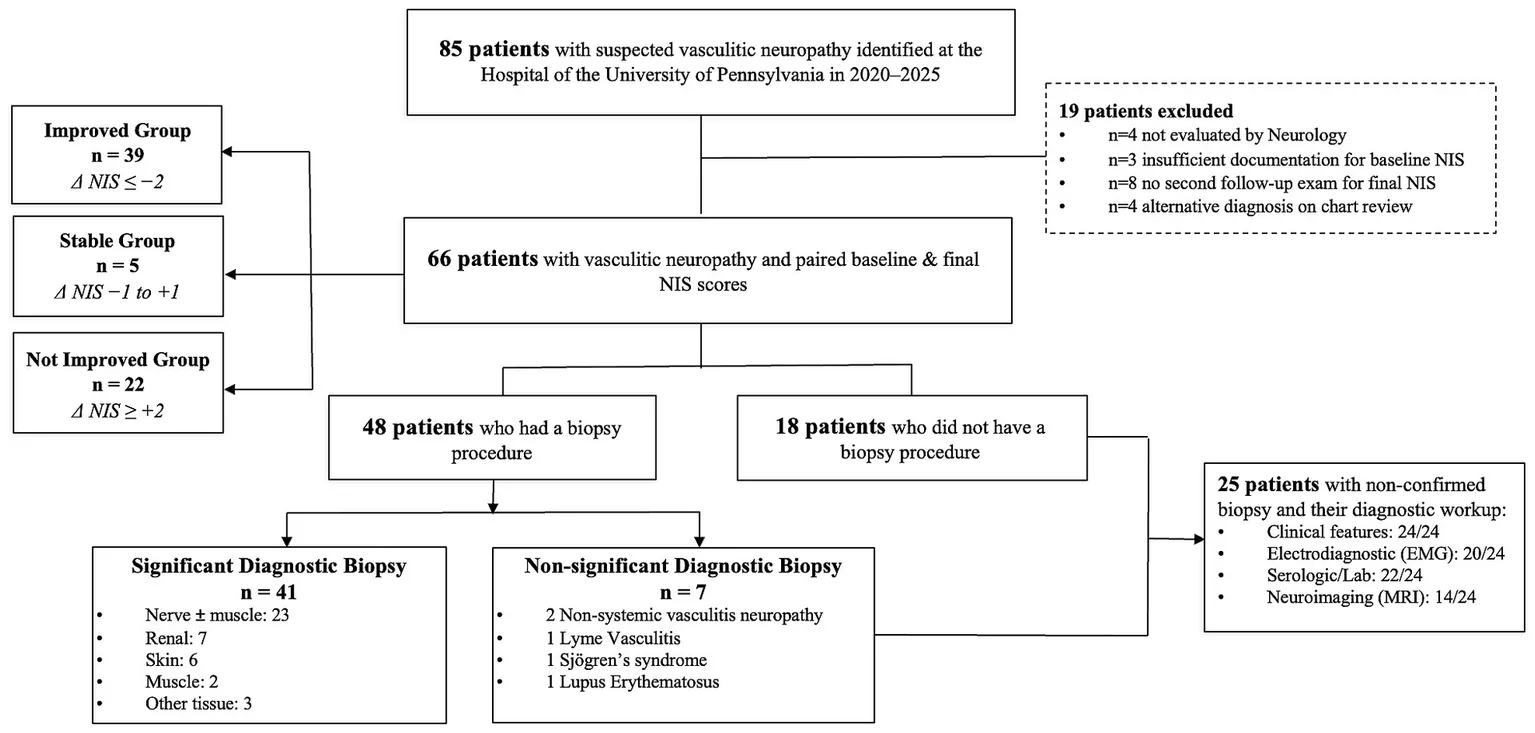
Study flow diagram. Eighty-five patients with suspected vasculitic neuropathy were identified at the Hospital of the University of Pennsylvania between October 2020 and December 2025; 19 were excluded, leaving 66 patients in the analytic cohort. Tissue biopsy was performed in 48 patients and showed vasculitis in 41 (biopsy-confirmed); 7 biopsies were non-diagnostic and 18 patients did not undergo biopsy. Patients without a confirmatory biopsy were diagnosed as possible vasculitic neuropathy using subtype-appropriate clinical, electrodiagnostic, and serologic criteria, including all 15 LRPN patients, in whom biopsy is not standard. Neurological outcomes were classified as Improved (ΔNIS ≤ − 2, *n* = 39), Stable (ΔNIS −1 to +1, *n* = 5), or Worsened (ΔNIS ≥ + 2, *n* = 22). The Stable and Worsened groups were combined as Not Improved (*n* = 27) for the primary analysis. NIS, Neuropathy Impairment Score.

Eighty-five adults with suspected VN were identified during the study period; 19 were excluded (4 not evaluated by Neurology, 3 with insufficient documentation to score a baseline NIS, 8 without follow-up assessment to score a final NIS, and 4 with an alternative diagnosis established on chart review), leaving 66 patients in the analytic cohort (Figure 1). The mean age at presentation was 64.4 ± 13.2 years, and 30 patients (45.5%) were women. Demographic and disease characteristics by neurological outcome are shown in Table 1. The cohort was equally divided between systemic vasculitic neuropathy (*n* = 33, 50.0%) and non-systemic vasculitic neuropathy (*n* = 33, 50.0%). The most common vasculitis subtypes were NSVN (*n* = 18, 27.3%), ANCA-associated vasculitis (*n* = 15, 22.7%), and diabetic lumbosacral radiculoplexus neuropathy (*n* = 15, 22.7%); cryoglobulinemic vasculitis (*n* = 7, 10.6%), polyarteritis nodosa (*n* = 5, 7.6%), and other connective tissue-associated vasculitides (Sjögren syndrome *n* = 3, systemic lupus erythematosus *n* = 2, Lyme-associated vasculitis *n* = 1) accounted for the remaining patients. Weakness was the most common presenting feature, documented in 59 of 66 patients (89.4%).

**Table 1:** Patient characteristics by neurological outcome (*N* = 66).

Characteristic	Overall *N* = 66	Improved *N* = 39	Not improved *N* = 27	*p*-value
Age, years	64.4 ± 13.2	62.0 ± 13.6	68.0 ± 11.9	0.062
Sex				
Female	30 (45.5%)	18 (46.2%)	12 (44.4%)	>0.999
Male	36 (54.5%)	21 (53.8%)	15 (55.6%)	
Vasculitis classification				
NSVN subtype	18 (27.3%)	14 (35.9%)	4 (14.8%)	0.092
LRPN (diabetic + non-diabetic)	15 (22.7%)	8 (20.5%)	7 (25.9%)	
Systemic VN	33 (50.0%)	17 (43.6%)	16 (59.3%)	
Vasculitis subtype				
NSVN subtype	18 (27.3%)	14 (35.9%)	4 (14.8%)	
ANCA-associated vasculitis	15 (22.7%)	9 (23.1%)	6 (22.2%)	
Diabetic LRPN	13 (19.7%)	7 (17.9%)	6 (22.2%)	
Non-diabetic LRPN	2 (3.0%)	1 (2.6%)	1 (3.7%)	
Cryoglobulinemic vasculitis	7 (10.6%)	3 (7.7%)	4 (14.8%)	
Polyarteritis nodosa	5 (7.6%)	3 (7.7%)	2 (7.4%)	
Sjögren-associated	3 (4.5%)	2 (5.1%)	1 (3.7%)	
Lupus-associated	2 (3.0%)	0 (0.0%)	2 (7.4%)	
Lyme-associated	1 (1.5%)	0 (0.0%)	1 (3.7%)	
Disease course				
Diagnostic delay, months	19.7 ± 30.5	12.3 ± 16.4	30.3 ± 41.7	0.042
Follow-up duration, months	20.7 ± 18.8	18.2 ± 19.0	24.3 ± 18.3	0.202
Baseline NIS subscores				
Baseline NIS, total	28.5 ± 19.5	34.5 ± 19.7	19.8 ± 15.9	0.001
NIS-motor	13.0 ± 13.3	17.3 ± 14.6	6.7 ± 7.9	<0.001
NIS-sensory	8.5 ± 7.1	9.5 ± 7.6	7.1 ± 6.1	0.155
NIS-reflex	6.7 ± 7.3	7.2 ± 6.3	6.1 ± 8.6	0.593
Treatment regimen				
Corticosteroid + steroid-sparing agent	34 (51.5%)	18 (46.2%)	16 (59.3%)	0.456
Corticosteroid monotherapy	19 (28.8%)	11 (28.2%)	8 (29.6%)	>0.999
IVIG-based (± steroid)	8 (12.1%)	7 (17.9%)	1 (3.7%)	0.130
Steroid-sparing agent alone	1 (1.5%)	1 (2.6%)	0 (0.0%)	>0.999
Supportive/no immunotherapy	4 (6.1%)	2 (5.1%)	2 (7.4%)	>0.999
Neuropathic pain agent	44 (66.7%)	28 (71.8%)	16 (59.3%)	0.421

Data are mean ± SD for continuous variables and *n* (%) for categorical variables.

Improved = ΔNIS ≤ − 2 from baseline to final assessment; Not Improved combines worsening (ΔNIS ≥ + 2, *n* = 22) and clinically stable (ΔNIS −1 to +1, *n* = 5) patients.

Non-systemic disease is reported as two distinct entities—the NSVN subtype and lumbosacral radiculoplexus neuropathy (LRPN)—to avoid the dual use of the term “NSVN.” Diagnostic certainty and biopsy confirmation are described in Figure 1.

Treatment regimens are mutually exclusive categories. Several regimen subgroups are small and the analysis is underpowered for formal comparison; *p*-values are descriptive.

Continuous variables compared with Welch *t*-test; categorical variables with Fisher exact test. No adjustment for multiple comparisons.

ANCA, antineutrophil cytoplasmic antibody; IVIG, intravenous immunoglobulin; LRPN, lumbosacral radiculoplexus neuropathy; NIS, Neuropathy Impairment Score; NSVN, nonsystemic vasculitic neuropathy; SD, standard deviation; VN, vasculitic neuropathy.

### Diagnostic classification and biopsy findings

In this cohort of 66 patients with vasculitic neuropathy, 48 underwent a tissue biopsy and 41 of these demonstrated vasculitis; 7 biopsies were non-diagnostic and 18 patients did not undergo biopsy. Among the 41 biopsy-confirmed cases, the diagnostic tissue was nerve with or without concurrent muscle in 23 patients (56.1%), kidney in 7 (17.1%), skin in 6 (14.6%), muscle alone in 2 (4.9%), and other tissue in 3 (7.3%). The 25 patients without biopsy confirmation comprised 18 who did not undergo biopsy and 7 with a non-diagnostic biopsy (2 NSVN subtype, 1 Lyme-associated, 1 Sjögren-associated, 1 lupus-associated, and 2 others). These patients were diagnosed as possible vasculitic neuropathy on subtype-appropriate clinical, electrodiagnostic, and serologic grounds. Notably, relying on nerve biopsy alone can yield false-negative results in roughly half of vasculitic neuropathy cases, owing to the patchy, segmental nature of vessel-wall inflammation; clinical, electrodiagnostic, and serologic criteria are therefore essential alongside histology (17, 19). Consistent with this, the two NSVN-subtype patients with a non-diagnostic biopsy (liver and muscle, respectively) were diagnosed on the basis of laboratory, clinical, and electrodiagnostic findings. All 15 LRPN patients were diagnosed on a characteristic lumbosacral radiculoplexus pattern on electromyography together with consistent clinical features and imaging, for whom nerve biopsy is neither required nor standard practice. Overall, 59 patients met criteria for definite and 7 for possible vasculitic neuropathy.

### Clinical manifestations and laboratory features

Clinical manifestations and laboratory findings are summarized in Table 2. Following complete re-extraction of clinical and serologic documentation (available in 66/66 patients), weakness (60/66, 90.9%) and pain (62/66, 93.9%) were near-universal and are reported here rather than tabulated. The most common remaining neurologic features were numbness (46/66, 69.7%) and foot drop (21/66, 31.8%). ANCA was positive in 15 of 66 patients (22.7%; 7 MPO-ANCA, 8 PR3-ANCA), cryoglobulins in 7 (10.6%), elevated erythrocyte sedimentation rate in 17 (25.8%), and elevated vascular endothelial growth factor (VEGF) in 5 (7.6%). Hypertension was documented in 25 (37.9%) and elevated glycated hemoglobin in 14 (21.2%), corresponding largely to the diabetic LRPN subgroup. Clinical manifestations and serologic results were documented in all 66 patients; electromyography reports were available in 28 of 66 and MRI in 14 of 66.

**Table 2:** Clinical manifestations and laboratory findings by neurological outcome (*N* = 66).

Characteristic	Overall *N* = 66	Improved *N* = 39	Not improved *N* = 27
Neurologic features			
Foot drop	21/66 (31.8%)	13/39 (33.3%)	8/27 (29.6%)
Gait impairment	4/66 (6.1%)	4/39 (10.3%)	0/27 (0.0%)
Numbness	46/66 (69.7%)	32/39 (82.1%)	14/27 (51.9%)
Extraneural manifestations			
Cutaneous ulcers	7/66 (10.6%)	5/39 (12.8%)	2/27 (7.4%)
Respiratory involvement	4/66 (6.1%)	3/39 (7.7%)	1/27 (3.7%)
Weight loss	6/66 (9.1%)	3/39 (7.7%)	3/27 (11.1%)
Hepatitis C	4/66 (6.1%)	2/39 (5.1%)	2/27 (7.4%)
Hypertension	25/66 (37.9%)	12/39 (30.8%)	13/27 (48.1%)
Autoantibody profile			
ANCA positivity (any)	15/66 (22.7%)	9/39 (23.1%)	6/27 (22.2%)
MPO-ANCA	7/66 (10.6%)	5/39 (12.8%)	2/27 (7.4%)
PR3-ANCA	8/66 (12.1%)	4/39 (10.3%)	4/27 (14.8%)
Cryoglobulins positive	7/66 (10.6%)	3/39 (7.7%)	4/27 (14.8%)
Elevated rheumatoid factor	5/66 (7.6%)	4/39 (10.3%)	1/27 (3.7%)
Elevated IgM/IgG	14/66 (21.2%)	9/39 (23.1%)	5/27 (18.5%)
Inflammatory markers and other laboratory findings			
Elevated ESR	17/66 (25.8%)	9/39 (23.1%)	8/27 (29.6%)
Elevated CRP	14/66 (21.2%)	10/39 (25.6%)	4/27 (14.8%)
Low complement C4	4/66 (6.1%)	3/39 (7.7%)	1/27 (3.7%)
Elevated CSF protein	7/66 (10.6%)	4/39 (10.3%)	3/27 (11.1%)
Diabetes (HbA1c documented)	14/66 (21.2%)	7/39 (17.9%)	7/27 (25.9%)
Elevated VEGF	5/66 (7.6%)	5/39 (12.8%)	0/27 (0.0%)
Elevated creatinine	7/66 (10.6%)	2/39 (5.1%)	5/27 (18.5%)
Elevated WBC	12/66 (18.2%)	7/39 (17.9%)	5/27 (18.5%)

Data are presented as *n*/*N* (%). Clinical manifestations and serologic/laboratory results were documented in all 66 patients. Weakness (60/66, 90.9%) and pain (62/66, 93.9%) were near-universal and are reported in the demographics text rather than tabulated. “Diabetes (HbA1c documented)” reflects patients with a documented elevated HbA1c and largely overlaps with the diabetic lumbosacral radiculoplexus neuropathy subgroup.

ANCA, antineutrophil cytoplasmic antibody; CRP, C-reactive protein; CSF, cerebrospinal fluid; ESR, erythrocyte sedimentation rate; HbA1c, glycated hemoglobin; IgG, immunoglobulin G; IgM, immunoglobulin M; MPO, myeloperoxidase; PR3, proteinase 3; VEGF, vascular endothelial growth factor; WBC, white blood cell count.

### Treatment

Treatment regimens are summarized in Table 1. Because immunotherapies overlapped substantially across patients, treatment was analyzed as mutually exclusive regimen categories rather than as single agents. The most common regimen was corticosteroid combined with a steroid-sparing agent (rituximab, cyclophosphamide, methotrexate, azathioprine, or mycophenolate) in 34 patients (51.5%), followed by corticosteroid monotherapy in 19 (28.8%), an intravenous immunoglobulin (IVIG)-based regimen with or without corticosteroid in 8 (12.1%), supportive care without immunotherapy in 4 (6.1%), and a steroid-sparing agent alone in 1 (1.5%). Symptomatic neuropathic pain agents (gabapentin, pregabalin, or duloxetine) were prescribed to 45 patients (68.2%). The distribution of treatment regimens did not differ significantly between outcome groups, although several regimen subgroups were small and the analysis was underpowered for formal comparison (Table 1).

## Neurological outcomes

**Figure 2:**
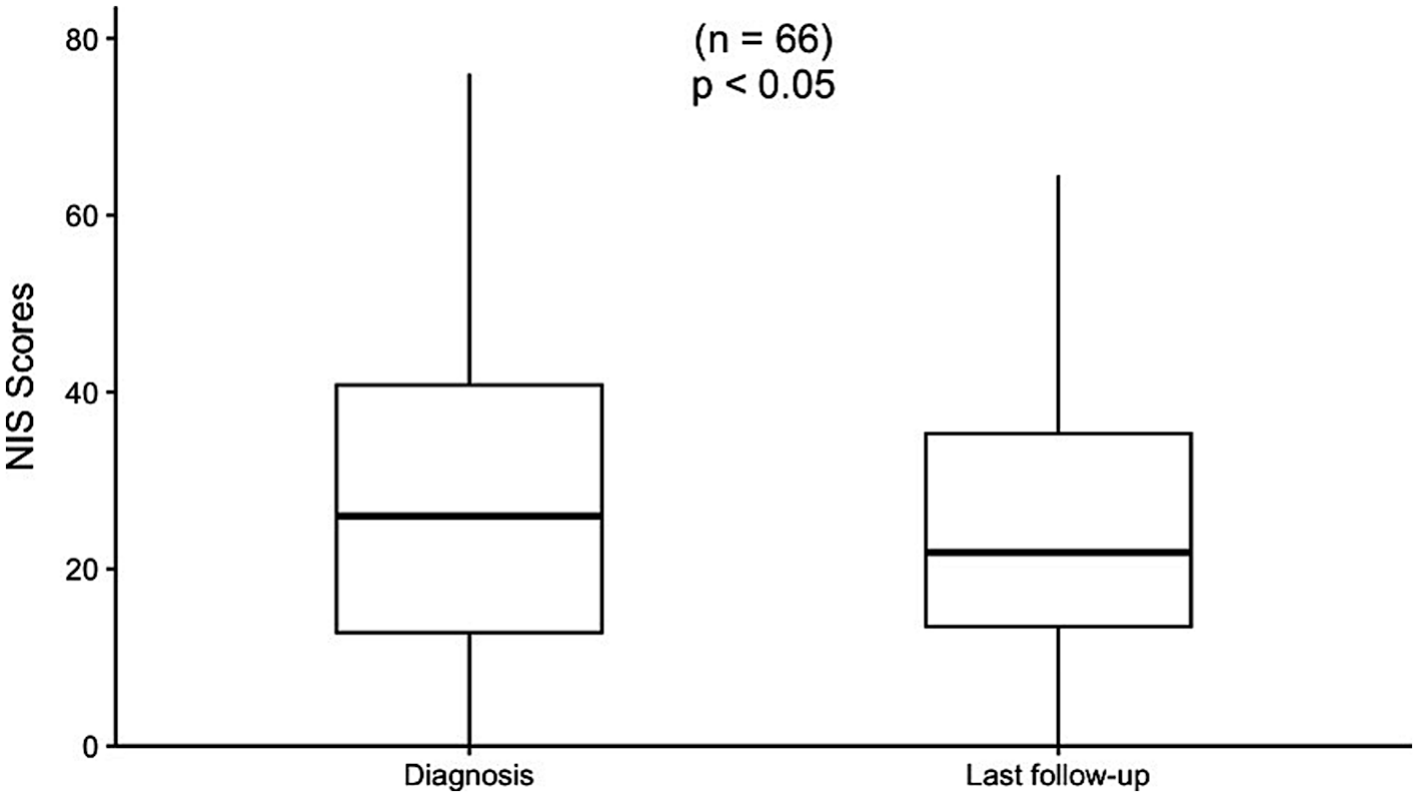
Distribution of Neuropathy Impairment Scores at diagnosis and at last follow-up. Side-by-side box plots showing the distribution of total NIS at diagnosis and at last follow-up across the analytic cohort (*N* = 66). Within-patient change between time points was assessed using the paired *t*-test.

Over a median follow-up of 13.5 months (interquartile range 6.2–31.2), 39 patients (59.1%) improved by ≥ 2 NIS points, 5 (7.6%) remained clinically stable (ΔNIS between −1 and +1 inclusive), and 22 (33.3%) worsened by ≥ 2 NIS points. The mean baseline NIS for the cohort was 28.5 ± 19.5 (range 0–76), and the mean final NIS was 25.7 ± 18.6. Figure 2 depicts the distribution of baseline and final NIS in the cohort.

### Exploratory factors associated with improvement

In exploratory unadjusted comparisons (Table 1), patients in the Improved group had higher baseline NIS-motor subscores (mean 17.3 ± 14.6 vs. 6.7 ± 7.9, *p* < 0.001) and shorter diagnostic delays (mean 12.3 ± 16.4 vs. 30.3 ± 41.7 months, *p* = 0.042). Improved patients also tended to be younger (mean 62.0 ± 13.6 vs. 68.0 ± 11.9 years, *p* = 0.062), although this difference did not reach statistical significance at the 0.05 threshold. Sensory and reflex NIS subscores, follow-up duration, vasculitis subtype, and vasculitis classification did not differ significantly between outcome groups (all *p* > 0.10). The distribution of neuropathy pattern differed between outcome groups in unadjusted analysis (*p* = 0.041), driven by a higher proportion of asymmetric polyneuropathy among Improved patients (23.1% vs. 3.7%) and a higher proportion of length-dependent polyneuropathy among Not Improved patients (40.7% vs. 23.1%).

Next, in order to explore whether specific phenotypes influenced diagnostic delay, patients with a multifocal or asymmetric phenotype had a numerically shorter median diagnostic delay than those with a length-dependent phenotype (8.1 vs. 19.5 months), but this difference was not statistically significant (*p* = 0.200). Similarly, patients without neuropathic pain had a longer median diagnostic delay than those with pain (42.0 vs. 8.1 months), though only 4 patients presented without pain and the difference was not significant (*p* = 0.164). These findings indicate that presenting phenotype and pain status were not significant determinants of diagnostic delay in this cohort, and are consistent with the multivariable analysis, in which adjustment for diagnostic delay did not alter the principal associations.

In an exploratory multivariable logistic regression model that included neuropathy pattern (multifocal vs. other), baseline NIS-motor subscore, age, vasculitis classification (systemic vs. non-systemic), and diagnostic delay, baseline NIS-motor subscore (adjusted odds ratio [OR] 1.08 per 1-point increase, 95% confidence interval [CI] 1.02–1.16, *p* = 0.020) and age (adjusted OR 0.95 per 1-year increase, 95% CI 0.90–1.00, *p* = 0.049) were independently associated with improvement. Neuropathy pattern (multifocal vs. other; adjusted OR 1.58, 95% CI 0.39–6.37, *p* = 0.522), vasculitis classification (systemic vs. non-systemic; adjusted OR 0.58, 95% CI 0.17–1.95, *p* = 0.379), and diagnostic delay (adjusted OR 0.98 per month, 95% CI 0.95–1.00, *p* = 0.126) were not independently associated with improvement after adjustment. Model calibration was good (Hosmer–Lemeshow goodness-of-fit *p* = 0.95) and discrimination was acceptable (area under the receiver operating characteristic curve 0.81, 95% CI 0.71–0.91). Variance inflation factors were all below 1.1, indicating no meaningful multicollinearity among predictors. Multivariable estimates are reported for descriptive purposes; the limited number of events per variable precludes their use as a clinical prediction tool.

### Lumbosacral radiculoplexus neuropathy subgroup

Of the 15 patients with diabetic lumbosacral radiculoplexus neuropathy, 11 (73.3%) received corticosteroids, 6 (40.0%) received intravenous immunoglobulin, and 13 (86.7%) received some form of immunomodulatory therapy; 2 (13.3%) were managed with symptomatic neuropathic pain therapy alone, and 14 (93.3%) received a neuropathic pain agent. Eight of the 15 (53.3%) improved by ≥ 2 NIS points over follow-up; treatment exposure did not differ between Improved and Not Improved DLRSN patients, consistent with the previously described natural history of typically self-limited microvasculitic injury in this entity (8, 10). The LRPN group comprised 13 diabetic and 2 non-diabetic patients; outcomes and treatment exposure were similar across these subgroups, although the non-diabetic group was too small (*n* = 2) for meaningful comparison.

## Discussion

In this single-center retrospective cohort of 66 adults with vasculitic neuropathy, more than half (59.1%) experienced neurological improvement on the NIS over a median follow-up of approximately 1 year, while one-third worsened despite contemporary immunomodulatory therapy. Greater baseline motor impairment and shorter diagnostic delay were associated with improvement in unadjusted analyses; baseline motor severity and younger age remained independently associated with improvement in a pre-specified multivariable model. Vasculitis subtype, neuropathy pattern, and treatment exposure did not differ between outcome groups after adjustment.

In the non-systemic spectrum, our results are broadly consistent with the French NSVN cohort reported by Quirin et al. (*n* = 50), in which over half of patients improved with immunosuppressive therapy (12). For diabetic and non-diabetic lumbosacral radiculoplexus neuropathy, our observation that DLRSN patients improved at similar rates regardless of treatment intensity aligns with the natural history described by Dyck et al., in whom partial recovery typically occurs over months as the inflammatory phase resolves (8–10). The role of immunosuppression in DLRSN therefore remains individualized rather than universally indicated (9).

The association between higher baseline motor severity and subsequent improvement is consistent with both a clinical observation and a biological rationale. Patients with greater baseline impairment have a greater absolute scope for measurable recovery before reaching the floor of the NIS, an effect sometimes described as regression to the mean. Beyond this measurement-driven explanation, focal ischemic axonal injury in vasculitic neuropathy is potentially recoverable through axonal regeneration and collateral reinnervation (18), whereas length-dependent axonal loss in long-standing or untreated disease may exceed the capacity for compensatory reinnervation. These mechanisms together may explain why patients presenting with greater (but reversible) motor deficits had a higher probability of measurable improvement than patients presenting with milder, more chronic, length-dependent disease.

It is worth noting that in unadjusted analysis the multifocal/mononeuritis multiplex pattern showed a higher proportion of improvers than other patterns, in keeping with traditional teaching of greater recoverability of multifocal vasculitic injury (2, 3). In our exploratory multivariable model, however, this association did not remain statistically significant after adjustment for baseline severity, age, vasculitis classification, and diagnostic delay (adjusted OR 1.58, *p* = 0.52). This stresses the importance of adjusting for disease classification and severity when interpreting pattern-based prognostic claims, as pooling systemic and non-systemic disease without accounting for baseline severity may produce strong pattern–outcome associations. Larger multicenter studies stratified by vasculitis class will be required to determine whether neuropathy phenotype carries independent prognostic information beyond severity.

Neuropathic pain was nearly universal in this cohort (93.9%). Although the small number of patients without pain (*n* = 4) precludes firm conclusions, their longer median diagnostic delay raises the possibility that painless presentations are recognized later, a hypothesis warranting study in larger cohorts.

Strengths of this study include the comprehensive application of the 2010 Peripheral Nerve Society and 2022 ACR/EULAR criteria for diagnostic classification, the use of the NIS as a validated quantitative outcome measure, the AANEM-accredited electrodiagnostic protocol, and the systematic reporting of denominators for variables with incomplete documentation. The cohort represents a contemporary North American tertiary-center experience that complements the predominantly European and Mayo Clinic-based VN literature.

This study has some limitations. First, it is a single-center retrospective study at a specialized tertiary neuromuscular referral center, so findings may not generalize to community settings or to populations with different referral patterns. Second, NIS scoring relied on chart-extracted examinations rather than standardized prospective assessment, and the interval between baseline and final NIS varied across patients. Third, the diagnostic workup was not uniform across the cohort. This reflects appropriate subtype-specific practice rather than a methodological shortcoming. Nerve or other tissue biopsy is central to confirming non-systemic vasculitic neuropathy and several systemic vasculitides, whereas diabetic lumbosacral radiculoplexus neuropathy is diagnosed on clinical and electrodiagnostic grounds, with biopsy neither required nor routinely pursued (4, 8, 10) Consistent with this, biopsy was not performed in most patients with diabetic lumbosacral radiculoplexus neuropathy, in whom the diagnosis was established by a characteristic electrodiagnostic pattern, clinical features, and supportive imaging; nerve biopsy in these patients would not have altered the diagnosis and is not standard practice. We classified patients using subtype-appropriate criteria (Peripheral Nerve Society 2010 and 2022 ACR/EULAR) and report diagnostic certainty transparently (Figure 1). Serologic and laboratory testing was obtained as clinically indicated rather than by fixed protocol, so denominators differ across laboratory variables (Table 2), and inference about infrequently ordered markers such as vascular endothelial growth factor and complement C3 is limited. Fourth, the sample size limits the precision of subgroup and multivariable estimates, so multivariable findings are reported as descriptive associations rather than as a validated prediction model (16). Fifth, treatment was not randomized and was individualized to vasculitis subtype and severity, which precludes causal inference about therapeutic effect.

Future research should prioritize prospective multicenter cohorts of VN with standardized examination intervals, uniform laboratory panels, and harmonized classification of neuropathy pattern, in order to validate whether baseline motor severity and diagnostic delay carry independent prognostic information beyond what is captured by vasculitis subtype and disease classification.

## Conclusion

In a contemporary single-center cohort of 66 adults with vasculitic neuropathy, the majority experienced neurological improvement on the NIS following individualized immunomodulatory treatment. Greater baseline motor impairment and shorter diagnostic delay were associated with improvement; vasculitis subtype, neuropathy pattern, and treatment exposure were not. These findings provide a benchmark for outcome expectations in contemporary tertiary-care VN practice and motivate larger, multicenter, prospective studies for confirmation.

## Data Availability

The raw data supporting the conclusions of this article will be made available by the authors, without undue reservation.
